# Gait disorders induced by photothrombotic cerebellar stroke in mice

**DOI:** 10.1038/s41598-023-42817-4

**Published:** 2023-09-22

**Authors:** Keisuke Inoue, Meiko Asaka, Sachiko Lee, Kinya Ishikawa, Dai Yanagihara

**Affiliations:** 1 Department of Life Sciences, Graduate School of Arts and Sciences, The University of Tokyo, Tokyo, Japan; 2 Department of Rehabilitation, JA Toride Medical Center, Toride, Japan; 3 Cognition and Behavior Joint Research Laboratory, RIKEN center for Brain Science, Wako, Japan; 4 Department of Rehabilitation Sciences, Graduate School of Medicine, Nagoya University, Nagoya, Japan; 5 The Center for Personalized Medicine for Healthy Aging, Tokyo Medical and Dental University, Tokyo, Japan; 6 Department of Neurology and Neurological Science, Graduate School of Medical and Sciences, Tokyo Medical and Dental University, Tokyo, Japan

**Keywords:** Neuroscience, Physiology, Neurology

## Abstract

Patients with cerebellar stroke display relatively mild ataxic gaits. These motor deficits often improve dramatically; however, the neural mechanisms of this improvement have yet to be elucidated. Previous studies in mouse models of gait ataxia, such as *ho15J* mice and *cbln1*-null mice, have shown that they have a dysfunction of parallel fiber-Purkinje cell synapses in the cerebellum. However, the effects of cerebellar stroke on the locomotor kinematics of wild-type mice are currently unknown. Here, we performed a kinematic analysis of gait ataxia caused by a photothrombotic stroke in the medial, vermal, and intermediate regions of the cerebellum of wild-type mice. We used the data and observations from this analysis to develop a model that will allow locomotive prognosis and indicate potential treatment regimens following a cerebellar stroke. Our analysis showed that mice performed poorly in a ladder rung test after a stroke. During walking on a treadmill, the mice with induced cerebellar stroke had an increased duty ratio of the hindlimb caused by shortened duration of the swing phase. Overall, our findings suggest that photothrombotic cerebellar infarction and kinematic gait analyses will provide a useful model for quantification of different types of acute management of cerebellar stroke in rodents.

## Introduction

Cerebellar stroke is one of the less common types of ischemic stroke^1^. Patients with cerebellar stroke have clinical symptoms that are characterized by postural dysfunctions^2^ and gait ataxia^2,3^, which are associated with increased risk of falls^2^. The postural dysfunctions are indicated by an increase in body sway or a higher postural score on the international cooperative ataxia rating scale (ICARS)^2^. Gait ataxia is displayed as a slower walking speed^3^, increased joint angle variability^3^, and a higher gait score on ICARS^2^. While these motor deficits often show remarkable improvements with time^2–4^, the essential mechanisms of improvement of the postural dysfunction and gait ataxia after cerebellar stroke have yet to be elucidated.

Various mouse models have been used to investigate cerebellar gait, namely, Purkinje cell degeneration (*pcd)* mice^5^, *reeler* mice^6^, and Lurcher mice^7,8^. Postnatally, *pcd* mice are characterized by the complete degeneration of the Purkinje cells and a subsequent partial loss of granule cells in the cerebellum^5^. *Reeler* mice are characterized by the aberrant localization of neurons and failure of neuronal layer formation in the cerebellum, hippocampus, neocortex, inferior olive, and substantia nigra^6^. Even if walking speed is corrected to that of wild-type mice, *pcd* and *reeler* mice display abnormally higher hind-paw trajectories during the swing phase and impaired inter-limb coordination during overground locomotion^5,6^. Additionally, *reeler* mice display increased vertical variability in fore-hindlimb displacement. Lurcher mice are characterized by a complete postnatal loss of Purkinje cells and a secondary retrograde degeneration of the cerebellar granule cells and inferior olivary neurons due to a mutation in the glutamate receptor delta2 (GluD2)-subunit gene, which is expressed predominantly by Purkinje cells^7^. Lurcher mice also display higher paw trajectories, increased vertical displacement of the hip, shortened stance duration, and abnormal inter-limb coordination of the hindlimbs during treadmill locomotion^8^. Although these mouse mutant models display apparently severe ataxic gait during overground locomotion^5–7^, some gait parameters do not differ from wild-type controls if walking speed is taken into account^5–7^. Therefore, in order to eliminate the influence of walking speed, it is necessary to analyze gait parameters on a treadmill^7^.

Our previous study examined hindlimb kinematic features during treadmill locomotion in *ho15J* mice^9^, which have an intragenic deletion in the gene encoding GluD2^9^. *ho15J* mice display excessive toe elevation during the swing phase, lower height of the great trochanter (GT), and severe hyperflexion of the ankles compared to wild-type mice^9^. In another study on *Cbln1*-null mice, which have a more severe ataxic gait than *ho15J* mice due to reduced parallel fiber (PF)-Purkinje cell (PC) synapses, it was found that the mice displayed shortened temporal parameters (stance duration, swing duration, and cycle time), excessive toe elevation during the swing phase, lower height of the GT, and severe hyperflexion of the ankles and knees compared to wild-type mice during treadmill locomotion^10^. These various types of mutant mice^5–10^ have abnormalities in their neural circuit architectures and in locomotion throughout growth and development; by contrast, in wild-type mice, a cerebellar stroke induces acute dysfunctions in gait from a previously healthy condition. The locomotor kinematics of cerebellar stroke are currently unknown.

Photothrombosis is one of the methods that can be used to induce focal ischemic strokes in an animal model; strokes are induced by generation of singlet oxygen species that lead to platelet activation and microvascular occlusion^11,12^. The principle of this method for inducing an infarct has closer similarities to human ischemic stroke than other methods such as intraluminal suture, middle cerebral artery occlusion, and endothelin 1 vasoconstriction^11^. Moreover, the photothrombotic stroke model has the advantages of low variability and mortality^11^, and can induce infarction in a reproducible manner in any targeted region that has a crucial role in coordinated and balanced locomotion, such as the cerebellar vermis and intermediate region^13^. The kinematic features of locomotion after a photothrombotic stroke in the cerebral cortex of mice were reported in a previous study^14^.

In the present study, we used kinematic analyses to investigate cerebellar gait ataxia following a photothrombotic stroke to the vermis and intermediate region of mice. We used our findings to develop a model that could be used for determining locomotive prognosis and for developing treatments following a cerebellar stroke.

## Results

**Figure 1:**
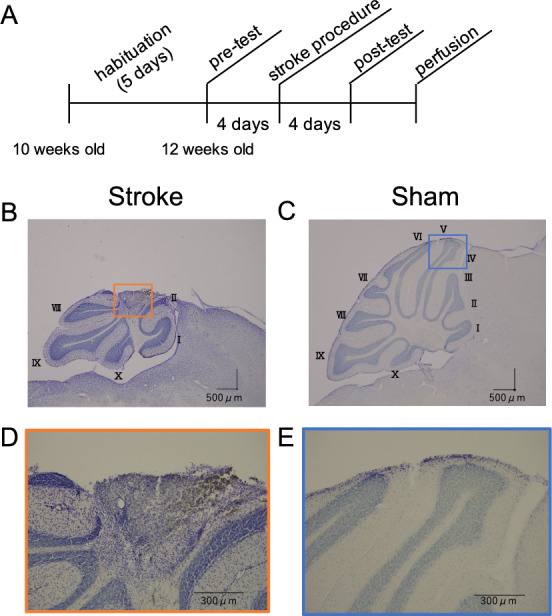
The experimental design and the histological verification of the cerebellar infarct region. The experimental design is shown in (**A**). Sagittal sections of the cerebellum after Nissl staining are shown for the stroke group (**B**,**D**) and the sham treatment group (**C**,**E**). Lobules numbers are indicated by Roman numerals. (**D**) and (**E**) are magnified images of the orange and blue squares in (**B**) and (**C**), respectively.

We performed the pre-test 4 days before the photothrombosis-induced cerebellar stroke (Fig. 1A). Two groups of experimental animals were established with similar mean body weights: stroke group, n = 8, mean body weight = 22.1 ± 0.9 g; sham treatment group, n = 6, mean body weight = 21.8 ± 0.9 g (mean ± standard deviation: SD). The focal infarct was induced in lobules IV and V in the cerebellar vermis; this region of the cerebellum is responsible for the control of limb movements during locomotion^15–17^. The post-test was performed 4 days after the photothrombotic stroke (Fig. 1A). Body weight was not affected by the cerebellar stroke (Supplementary Table S1 online). However, two mice in the stroke group were excluded from further analyses: one was excluded because the laser probe moved from the midline during illumination with the green laser; the second was excluded because of significant asymmetry of the step cycle time during treadmill walking in the pre-test.

The results of the histological analyses are shown in Fig. 1B–E. Serial sagittal sections confirmed that the infarct region was located in lobules IV, V and VI, in which the molecular, Purkinje cell, and granule cell layers were identified to be damaged and was restricted to the relatively superficial region of the cerebellar cortex in all six animals (Fig. 1B,D). In addition, the infarct region affected the vermis and the bilateral medial side of the intermediate region (right: 1520 ± 281 μm; left: 1373 ± 224 μm; mean ± SD).

**Figure 2:**
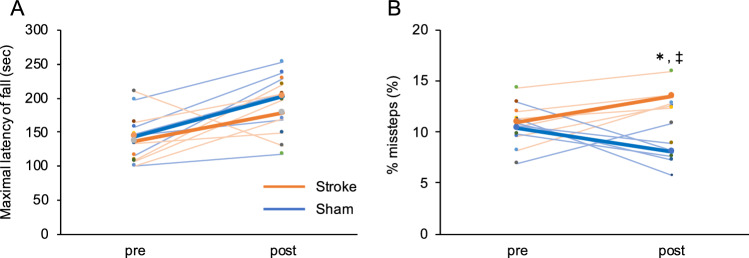
The results of the accelerating rotarod test and the ladder rung test. (**A**) Maximal latency of fall in the accelerating rotarod test. (**B**) Rate of missteps in the ladder rung test. Orange and blue lines indicate stroke and sham treatment groups, respectively. The thin and thick lines indicate individual and mean data respectively. Data were analyzed by two-way repeated-measures ANOVA with post-hoc Bonferroni test. *pre-test vs post-test, p < 0.05. ^‡^sham vs stroke, p < 0.01.

The maximal latency of fall in the accelerating rotarod test (Fig. 2A, Supplementary Table S1 online) showed significant pre-post effects (F (1, 10) = 9.879, p = 0.010), but no significant group effects (F (1, 10) = 0.738, p = 0.410) or interaction (F (1, 10) = 0.274, p = 0.612). In the ladder rung test (Supplementary Fig. S2 online), the rate (%) of missteps (number of missteps/ number of steps) did not show significant pre-post effects (F (1, 10) = 0.010, p = 0.923), but did show significant group effects (F (1, 10) = 16.205, p = 0.022) and interaction (F (1, 10) = 10.495, p = 0.009). A post hoc statistical test identified a significant increase in the rate of missteps in the stroke group (pre-test, 11.0 ± 2.1%; post-test, 13.5 ± 1.3%, p = 0.040; mean ± SD); the sham treatment group showed a trend of decreasing missteps (pre-test, 10.4 ± 2.0%; post-test, 8.1 ± 1.7%, p = 0.051; mean ± SD), whereas the stroke group had a higher rate of missteps compared to the sham treatment group at the post-test (p < 0.001) (Fig. 2B, Supplementary Table S1 online). Thus, the rate of missteps in the ladder rung test was significantly affected by the induced cerebellar stroke.

**Figure 3:**
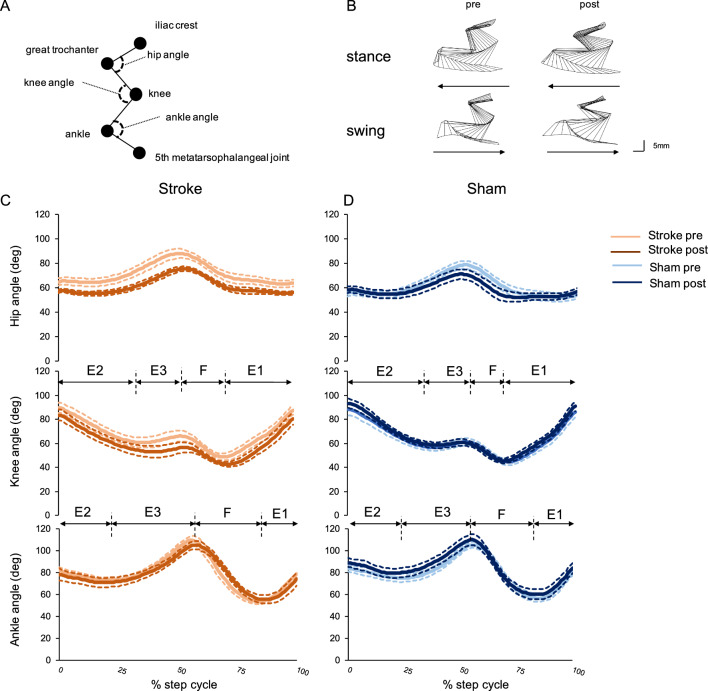
Kinematic analyses in the treadmill walking test. (**A**) Positions of the reflective markers. Stick figures show representative right hindlimb trajectories during stance and swing phases in the stroke group (**B**). Hip, knee, and ankle angular displacement during a normalized step cycle in the stroke group (**C**) and sham treatment group (**D**). Orange and brown lines indicate pre- and post-test in the stroke group, respectively. Light blue and blue lines indicate pre- and post-test in the sham treatment group, respectively. Dotted lines indicate SEM.

**Figure 4:**
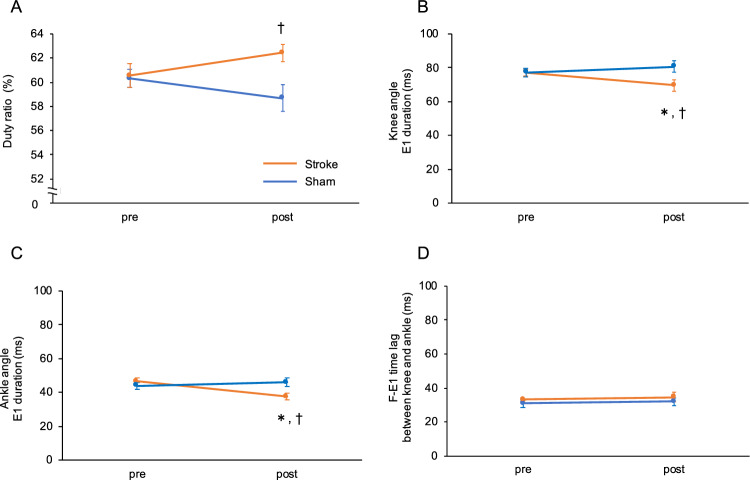
Temporal parameters in the treadmill walking test. Data were analyzed by two-way repeated-measures ANOVA with a post-hoc Bonferroni test (**A**–**D**). Duty ratio (**A**), E1 duration of knee (**B**), E1 duration of ankle (**C**), and the F-E1 time lag between knee and ankle joints (**D**). Orange and blue lines indicate stroke and sham treatment groups, respectively. *pre-test vs post- test, p < 0.05. ^†^sham vs stroke, p < 0.05. Error bar indicates SEM.

The positions of the markers on the joints analyzed in the treadmill walking test are illustrated in Fig. 3A. The stick figures show the movements of the right hindlimb in the stance and swing phases separately during a single step cycle (Fig. 3B). Hip, knee, and ankle angle displacement in the stroke and sham treatment groups are shown in Fig. 3C and D, respectively. The duty ratio of the right hindlimb did not have pre-post effects (F (1, 10) = 0.034, p = 0.857) or group effects (F (1, 10) = 3.517, p = 0.090), but did show significant interaction (F (1, 10) = 6.030, p = 0.034; Supplementary Table S3 online). In the post hoc test, the duty ratio of the stroke group was significantly higher than that of the sham treatment group (stroke = 62.4 ± 0.7%; sham treatment = 58.7 ± 1.1%, p = 0.017) (Fig. 4A). The duration of the F phase of knee joints did not show pre-post effects (F (1, 10) = 0.721, p = 0.416) or group effects (F (1, 10) = 0.041, p = 0.844), but did show a significant interaction (F (1, 10) = 5.883, p = 0.036). In the post hoc test, the duration of the F phase of the stroke group was significantly shortened (pre = 29.8 ± 1.7 ms; post = 27.0 ± 0.8 ms, p = 0.043). However, because the reduction in time (< 5 ms) was less than the temporal resolution of our motion capture system (200 frames per second using six high-speed digital image cameras), we were unable to conclude whether or not these changes in duration of the F phase were meaningful. The F phase durations of the ankle joints did not show any effects of the induced stroke for pre-post effects (F (1, 10) = 0.042, p = 0.841), group effects (F (1, 10) = 0.039, p = 0.848), or interaction (F (1, 10) = 0.162, p = 0.696). The duration of the E1 phase of the knee joint did not show pre-post effects (F (1, 10) = 0.904, p = 0.364) or group effects (F (1, 10) = 2.771, p = 0.127), but did show a significant interaction (F (1, 10) = 6.908, p = 0.025). In the post hoc test, the E1 phase of the knee joint in the stroke group was significantly reduced (pre = 77.2 ± 2.1 ms; post = 69.5 ± 3.1 ms, p = 0.030) compared to the sham treatment group (stroke group = 69.5 ± 3.1 ms; sham treatment group = 80.8 ± 3.5 ms, p = 0.037) at the post-test (Fig. 4B,D). Similarly, the duration of the E1 phase of the ankle joint did not show pre-post effects (F (1, 10) = 3.162, p = 0.106) or group effects (F (1, 10) = 1.557, p = 0.241), but did have a significant interaction (F (1, 10) = 7.981, p = 0.018). In the post hoc test, the duration of the E1 phase of the ankle joint in the stroke group was significantly reduced (pre = 46.4 ± 2.1 ms; post = 37.5 ± 2.0 ms, p = 0.009) compared to the sham treatment group (stroke = 37.5 ± 2.0 ms; sham treatment = 45.9 ± 2.5 ms, p = 0.026) at the post-test (Fig. 4C,D). The F-E1 time lag between the knee and ankle joints (F-E1 time of knee—ankle) did not show any effects in the pre-post test (F (1, 10) = 0.382, p = 0.550), group test (F (1, 10) = 1.577, p = 0.238), or interaction (F (1, 10) = 0.008, p = 0.930). These data indicated that the higher duty ratio in the stroke group was mainly caused by changes in the durations of the swing phase, particularly by the shortened E1 phase.

In contrast to previous studies in ataxic mouse models, we found here that maximal metatarsophalangeal (MTP) heights^5,6,8–10^ representing ankle assessment, average GT heights^8–10^, and the coefficient of variation (CV) of the MTP displacements representing toe assessment^7^ were not significantly altered after induced stroke (Table 1). However, knee angle at the post-test tended to show greater flexion than that at the pre-test during the step cycle (Fig. 3C) in the stroke group; in particular, knee angle at E3-F and F-E1 showed hyperflexion compared to the pre-test (E3-F pre = 70.8 ± 4.4 deg; E3-F post = 60.2 ± 3.9 deg, p = 0.027; F-E1 pre = 45.2 ± 2.8 deg; F-E1 post = 38.1 ± 2.4 deg, p = 0.026), although the interaction of both E3-F and F-E1 knee angles did not reach statistical significance (Table 1). The ankle angle did not have any effects (Table 1).

**Table 1:** Numerical and statistical data for hindlimb spatial parameters in the treadmill walking test.

			Pre-test	Pre-test	Two-way ANOVA		
					Pre-post	Group	Interaction
Maximal MTP height (mm)		Stroke	12.1 ± 0.6	12.9 ± 1.2	F(1,10) = 0.214, p = 0.654	F(1,10) = 0.000, p = 0.999	F(1,10) = 0.995, p = 0.342
		Sham	12.6 ± 0.8	12.3 ± 0.6			
Average GT height (mm)		Stroke	26.0 ± 1.3	25.6 ± 0.8	F(1,10) = 0.548, p = 0.476	F(1,10) = 0.151, p = 0.706	F(1,10) = 1.834, p = 0.205
		Sham	25.6 ± 0.9	27.0 ± 0.9			
CV vertical MTP displacement (%)		Stroke	9.8 ± 0.7	11.3 ± 2.2	F(1,10) = 0.896, p = 0.366	F(1,10) = 0.515, p = 0.489	F(1,10) = 0.044, p = 0.838
		Sham	9.2 ± 1.1	10.2 ± 0.6			
Hip angle (deg)	Foot contact	Stroke	64.9 ± 2.9^†^	57.2 ± 1.5*	F(1,10) = 1.242, p = 0.291	F(1,10) = 2.511, p = 0.144	F(1,10) = 5.813, p = 0.037
		Sham	55.2 ± 2.6	58.1 ± 2.7*			
	Foot lift	Stroke	80.7 ± 3.9	68.8 ± 2.0	F(1,10) = 9.844, p = 0.011	F(1,10) = 3.985, p = 0.074	F(1,10) = 0.846, p = 0.379
		Sham	70.1 ± 3.1	63.6 ± 4.4			
Knee angle (deg)	Foot contact	Stroke	89.9 ± 4.2	83.6 ± 4.6	F(1,10) = 0.051, p = 0.826	F(1,10) = 0.640, p = 0.442	F(1,10) = 2.885, p = 0.120
		Sham	88.5 ± 4.9	93.4 ± 3.9			
	Foot lift	Stroke	57.8 ± 4.2	49.2 ± 2.5	F(1,10) = 2.018, p = 0.186	F(1,10) = 0.004, p = 0.952	F(1,10) = 2.124, p = 0.176
		Sham	53.7 ± 4.2	53.8 ± 2.9			
	E3-F1	Stroke	70.8 ± 4.4	60.2 ± 3.9*	F(1,10) = 5.044, p = 0.049	F(1,10) = 1.988, p = 0.189	F(1,10) = 1.988, p = 0.189
		Sham	65.4 ± 3.6	63.0 ± 2.8			
	F-E1	Stroke	45.2 ± 2.8	38.1 ± 2.4*	F(1,10) = 2.649, p = 0.135	F(1,10) = 0.145, p = 0.711	F(1,10) = 4.246, p = 0.066
		Sham	40.2 ± 2.3	41.1 ± 1.7			
Ankle angle (deg)	Foot contact	Stroke	82.0 ± 2.9	78.5 ± 5.5	F(1,10) = 0.052, p = 0.825	F(1,10) = 1.784, p = 0.211	F(1,10) = 2.176, p = 0.171
		Sham	84.6 ± 3.1	89.3 ± 4.2			
	Foot lift	Stroke	108.5 ± 3.0	107.9 ± 4.4	F(1,10) = 0.163, p = 0.695	F(1,10) = 0.361, p = 0.561	F(1,10) = 0.295, p = 0.599
		Sham	108.3 ± 1.3	112.1 ± 4.9			
	E3-F1	Stroke	119.8 ± 3.7	116.9 ± 4.0	F(1,10) = 0.010, p = 0.921	F(1,10) = 0.108, p = 0.750	F(1,10) = 0.359, p = 0.563
		Sham	118.5 ± 2.8	120.6 ± 4.7			
	F-E1	Stroke	51.5 ± 2.6	53.6 ± 3.7	F(1,10) = 0.647, p = 0.440	F(1,10) = 1.052, p = 0.329	F(1,10) = 0.037, p = 0.852
		Sham	55.0 ± 3.4	58.4 ± 4.8			

Data were analyzed by two-way repeated-measures ANOVA with a post-hoc Bonferroni test. Data are shown as means ± SEM from six animals in each group.

*pre-test vs post-test, p < 0.05. ^†^sham vs stroke, p < 0.05.

**Figure 5:**
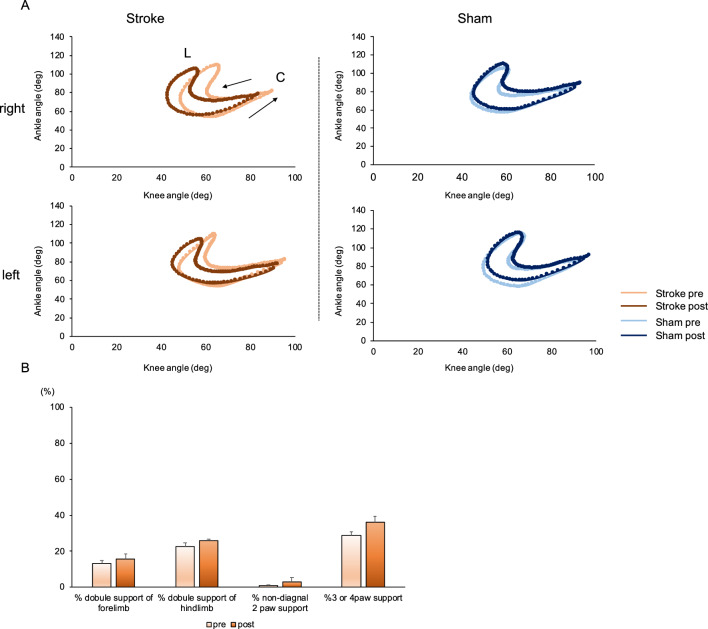
Intra-limb coordination and paw support patterns. (**A**) Intra-limb coordination of the knee and ankle joints during the step cycle in the stroke group (left) and sham treatment group (right). The upper diagram shows the right hindlimb and the lower diagram shows the left hindlimb. C: Foot contact at the beginning of the stance phase. L: foot lift at the beginning of the swing phase. Arrows represent the direction of angular motion. (**B**) Changes in rates of paw support patterns are shown relative to stance duration in the stroke group (pre, orange; post, brown). Rates of double support by a forelimb and a hindlimb; rates of non-diagonal 2-paw support and rates of 3- or 4-paw support were not affected by the induced cerebellar stroke. Data were analyzed by paired-t test or Wilcoxon signed-rank test. Error bar represents SEM.

We compared intra-limb coordination of the knee and ankle joints in the two groups of mice (Fig. 5A). In both the stroke and sham treatment groups, knee-ankle movements described a crescent shape, as has been reported previously in wild-type mice^9,10^. However, knee-ankle movements in the two groups of mice did show some differences. Thus, mice in the induced stroke group showed hyperflexion of the knee joint during the step cycle (Fig. 3C), although this change to knee-ankle movement was less than that described for *ho15J* and *cbln1*-null mice^9,10^.

Paw support patterns in the stroke group are shown in Fig. 5B. Although *pcd* mice^5^ and *reeler* mice^6^ have higher rates of forelimb double support configurations and lower rates of hindlimb double support configurations than wild-type mice, the stroke group in the present study did not show significant changes for either forelimb (pre = 13.2 ± 1.6%; post = 15.8 ± 2.6%, p = 0.301) or hindlimb (pre = 22.7 ± 2.0%; post = 25.7 ± 1.1%, p = 0.083) double support configurations. In addition, the rates of 3- or 4-paw support were not significantly increased: pre = 28.7 ± 1.9%; post = 36.3 ± 3.2%, p = 0.094. Non-diagonal 2-paw support did not show any changes: pre = 0.9 ± 0.5%; post = 3.6 ± 2.6%, p = 0.686). These observations differ from those reported for *pcd* mice^5^ and *reeler* mice^6^. From these data, we conclude that intra-limb coordination and paw support patterns in the stroke group were not as severely affected as those of mice carrying mutations for genes involved in cerebellar function^5–10^.

## Discussion

In the present study, localized infarction was induced by photothrombosis in the vermal and intermediate regions; lobules IV, V, and VI were affected in all treated animals. We found that the rates of missteps in a ladder rung test were increased by the induced cerebellar stroke, although the behavior of the mice in an accelerating rotarod test was not significantly affected. During walking on a treadmill, the duty ratio of the hindlimbs of mice in the stroke group was higher than that in the sham treatment group; the increased duty ratio resulted from a shortened duration of the E1 phase during swing movements. Moreover, knee angles at E3-F and F-E1 were decreased in the mice in the cerebellar stroke group, although hind-paw trajectories during the swing phase and vertical displacement of hip were not affected.

In an accelerating rotarod test, the mice in the stroke group had a similar retention time as the sham treatment group. Rotarod tests are often used to assess ataxic gait due to cerebellar damage^18–20^. Mice homozygous for a null mutation of the metabotropic glutamate receptor-subtype 1 (mGluR1) gene^18^ and spinocerebellar ataxia type 1 (SCA1) model mice^19^ have functional impairments of mGluR signaling throughout the cerebellum; both types of mice display poor performance in rotarod tests. However, mice with electrolytically-induced lesions in the bilateral intermediate region do not show poor performance in rotarod tests^20^. In the present study, the lesions induced in the vermis were comparatively small and localized and this may be the reason for the lack of gait disorders in the rotarod test. Additionally, Takeuchi et al.^10^ suggested that knee-ankle coordination might affect retention time in rotarod tests. In the present study, knee-ankle intra-limb coordination showed milder impairment than in *ho15J* mice^9^ and *cbln1*-null mice^10^. We speculate that the non-infarcted regions of the cerebellum might compensate for the neural functions of the lesioned region and result in the relatively mild ataxic gait of the treated mice^21^.

We found that mice in the stroke group showed an increased rate of missteps in a ladder rung test. It has been reported that *pcd* mice display more frequent missteps than wild-type mice^22^. Lesions to intermediate anterior lobules or interposed nuclei in cats were suggested to cause difficulty in ladder rung tests^23^. The intermediate region was proposed to have a crucial role in directing limb placement and regulating agonist–antagonist muscle pairs, especially in circumstances where greater precision was required^13^. Therefore, it is possible that the increased rate of missteps in this study might have been caused by lesions within the intermediate region.

The treadmill walking test indicated that mice with induced cerebellar stroke had an increased duty ratio in the right hindlimb caused by a shortened E1 duration and a decreased right knee angle at E3-F and F-E1. Both temporal and spatial changes were observed on the right side. This laterality might have been caused by the infarction being more extended to the right side than the left side as suggested by our histological analyses.

With regard to temporal parameters, a shortened swing duration was reported in a study on *cbln1*-null mice^10^. As E1 duration describes the time just before foot contact, it has been suggested that a shortened E1 duration might affect goal-directed movement. This may explain why the rate of missteps in the ladder rung test here might have been affected in mice with induced cerebellar stroke. In spatial parameters, we found a tendency for decreased knee angles during the step cycle. Similar observations have been reported in studies on the inactivation of the anterior intermediate region in rats^24^ and in *cbln1*-null mice^10^. In addition, our study also observed a decreased knee angle at E3-F and F-E1 in mice of the stroke group. In a study on decerebrate cats, Udo et al. showed that unilateral partial cooling of the cerebellar cortex at lobules V of the intermediate region induced hyperflexion of the ipsilateral forelimb^25,26^, while cooling of lobules IV of the intermediate region induced hyperflexion of the ipsilateral hip and ankle^25^. Taken together, these reports indicate that the cerebellar cortex is involved in the activity of limb muscles and that E1 duration is modulated by flexor and extensor muscle activities^25^. Moreover, it has been shown that simple spike discharges from lobules V and VI of the cerebellar vermis are highest in the E3 or F and E1 phases and are related to the onset of flexor and extensor activities on the ipsilateral hindlimb^15^. From an anatomical point of view, the cerebellar neural circuit participates in the control of locomotion as part of the spinocerebellar loop^13^. Therefore, it is possible that the observed temporal and spatial changes during walking on the treadmill were mainly induced by the loss of Purkinje neurons in the vermis and intermediate region. However, maximal MTP heights^5,6,8–10^, the CV of vertical toe displacement^6^, vertical displacement of the hip^8–10^, and paw support patterns^5,6,8^ were not affected by the induced cerebellar stroke in contrast to previous studies in mutant mice. As mentioned above, it is possible that the relative lack of impairment in mice with an induced stroke during walking on the treadmill compared with mutant mice is due to the localized infarction in the cerebellum.

We identified several similarities between mice with induced cerebellar stroke and human patients with acerebellar stroke. First, in humans, gait ataxia caused by the cerebellar infarction often shows remarkable improvement from the acute phase such that gait disorders and activities are relatively milder at the post-acute phase^2,4^. The mice in our study displayed milder gait disorders compared to mouse models of cerebellar damage^5–10^. Second, ataxic gait in patients is associated with an increased risk of falling^2^; mice in our induced stroke group showed an increased rate of missteps in the ladder rung test. Finally, patients with lesions in the intermediate region show impaired goal-directed movement when walking^27^; the mice in our induced stroke group showed a shortened E1 duration which may indicate deficiency in goal-directed movement.

We conclude that although gait disorders induced by photothrombotic stroke are milder than those found in mutant mice^5–10^, nevertheless, kinematic changes are observed in a ladder rung test and during treadmill locomotion. These results suggest that photothrombotic cerebellar infarction and kinematic gait analyses will provide a useful model for quantification of different strategies for acute management of cerebellar stroke in rodents.

## Methods

### Animals

Experiments were performed on C57BL/6 J mice (n = 14, male, 10 weeks old) purchased from CREA Japan, Inc (Tokyo, Japan). The animals were kept in a temperature-controlled room with 12-h light/dark cycle (light on from 19:00 to 7:00). They were provided with food and water ad lib and housed individually in standard cages. The well-being of the mice was carefully monitored and all efforts were made to minimize the number of animals used and any suffering in the course of the experiments. This study was approved by the Ethical Committee for Animal Experiments at the University of Tokyo, and was carried out in accordance with the Guidelines for Research with Experimental Animals of the University of Tokyo and the Guide for the Care and Use of Laboratory Animals (NIH Guide, revised in 1996). This study was carried out in compliance with the ARRIVE guidelines.

### Locomotion tests

In the accelerating rotarod test, the mice were habituated to the rotarod apparatus (Rota-rod treadmill for mice MK-610A, Muromachi Kikai Co., Ltd, Tokyo, Japan) and trained to remain on the rod (from 2 to 20 rpm, 5 min) prior to the recordings. The protocol of the rotarod test involved acceleration from 4 to 40 rpm over 5 min; the rotarod was stopped at 300 s if the mice successfully stayed on for this duration. The mice performed in 4 tests and rested for 30 min between each test. We measured the maximal latency of fall in the 4 tests.

In the ladder rung test, video recordings were made of mice walking on a horizontal ladder (Supplementary Fig. S2 online; length: 40 cm width: 6 cm) with an irregular arrangement of rungs (7.5–15 mm; Custom order, O’HARA & Co., Ltd, Tokyo, Japan). We measured the total number of steps and the number of missteps during 10 crossings; the rate of missteps was calculated as: number of missteps/total number of steps.

To enable observation of hindlimbs in the treadmill walking test, the fur on the hindlimb was shaved under isoflurane gas anesthesia (3% for induction, 1–2% maintenance). Circular reflective markers (2 mm diameter) were precisely placed on the shaved skin of the hindlimb at the iliac crest, the great trochanter (GT), the knee, the ankle, and the 5th metatarsophalangeal (MTP) joint. The mice were allowed to recover completely from the anesthesia before the treadmill walking test. The animals walked freely at 20 m/min imposed by the treadmill belt (length: 17.5 cm, width: 6.5 cm, height: 12 cm; Custom order, O’HARA & Co., Ltd, Tokyo, Japan) and their locomotor movements were recorded at 200 frames per second using six high-speed digital image cameras (HAS-220 and HAS-I1, DITECT, Inc., Tokyo, Japan). The captured images were stored electronically for later analysis.

### Motion analysis of the hindlimbs in the treadmill walking test

Motion analysis software (DIPP-Motion V/3D, DITECT, Inc., Tokyo, Japan) was used to extract the three-dimensional coordinates of the various joint markers and to reconstruct a stick diagram representation of both hindlimbs. Due to skin slippage above the knee joint during walking, the actual knee position was corrected by triangulation from the position of the hip and ankle joint, using the measured length of the femur and tibia (MATLAB, Math Works, Inc., USA). In present study, we analyzed the data from at least nine steps cycles and excluded data when neither foot was on the treadmill. Then, we performed kinematic analysis with temporal (stance and swing duration, a step cycle time, and duty ratio) and spatial parameters (the position of markers and the angle of each joint). The stance phase was defined as starting when the foot contacted the treadmill belt and as ending when the foot lifted from the belt. The swing phase was defined as starting at the moment when the mice lifted its foot from the treadmill belt and as ending just before the foot contacted the belts. The duty ratio was calculated as: stance duration/a step cycle time. The angular displacements of the knee and ankle joints were divided into a flexion (F) and three extension (E1, E2 and E3) phases (Fig. 3C). The transition point from extension to flexion is E3-F. The transition point from flexion to extension during the swing phase is F-E1. To analyze the angular displacement of hip, knee, and ankle during a step cycle, the step cycle duration was normalized using 100 samples per step cycle.

### Stroke procedures

The mice were divided into two groups with similar mean body weights (stroke, n = 8, 22.1 ± 0.3 g; sham, n = 6, 21.8 ± 0.3 g). Mice in the induced stroke group were confined in a stereotaxic apparatus (SR-6 M-HT, NARISHIGE Group, Tokyo, Japan) and anesthetized using isoflurane gas (3% for induction, 1–2% for maintenance; FUJIFILM Wako Pure Chemical Corporation, Osaka, Japan). The occipital skin was incised and the occipital bone, 1 mm caudal to lambda, was drilled through to the dura (URAWA Corporation CO., Ltd, Saitama, Japan). We minimized the stress to the mice by keeping them warm; we monitored rectal temperatures and heart rates. Rose Bengal dye (30 μg/ g body weight; Sigma-Aldrich) was injected via the tail vein. Then, a 1 mm diameter probe was placed over the surface of the drilled region, the location of lobules IV and V, for illumination by a green laser (532 nm, 100 mW, 10 min; MGL-III-532-100mW, Changchun New Industries Optoelectronics Technology CO., Ltd, China). After illumination, the occipital bone was covered by bonewax and the skin was sutured. Sham animals were submitted to the same surgical procedure as described above, but without laser illumination. The mice were given a 4-day recovery period prior to locomotion tests. This interval was previously shown to be after the peak of expansion of cerebellar infarction induced by photothrombotic ischemia^28^.

### Histology

The mice were sacrificed under deep anesthesia (5% isoflurane gas) and perfused transcardially with saline (16 ml/min, 5 min) and 4% paraformaldehyde (PFA) in 0.1 M phosphate buffer (PB) (16 ml/ min, 5 min). After perfusion, the extracted brain was postfixed for more than 7 days in the same fixative; the extracted brain was then sequentially immersed in 5, 10, and 20% sucrose in 0.1 M PB for 3 days at each concentration.

After fixation, serial sagittal Sections (40 μm) of the cerebellum were prepared with a freezing microtome (REM-700, YAMATO KOHKI INDUSTRIAL CO., Ltd, Japan); the sections were collected in vials containing 0.1 M PB, and then mounted on glass microscope slides and fully dried. The sections were Nissl stained with cresyl violet. We analyzed the lesioned lobules and the extent of the infarct region (left–right diameter) by a digital microscope (BZ-X710, Keyence, Japan).

### Statistical analysis

Statistical analyses were performed using standard statistical software (SPSS Statistics version 25, IBM, Japan). All data were tested for normal distribution using the Shapiro–Wilk test. Normally distributed data were analyzed by two-way repeated-measures analysis of variance (ANOVA). In a post-hoc test, we applied the Bonferroni correction. We also compared pre-test data with post-test data in the stroke group using a paired-t test. Nonparametric data were tested using a Wilcoxon signed-rank test. The level of statistical significance for variables was set at P < 0.05. Data on stroke area size, body weight, accelerating rotarod test, and ladder rung test are expressed as means ± SD. Data from the treadmill walking test are expressed as mean ± standard error of the mean (SEM).

### Supplementary Information

Supplementary Information 1.

Supplementary Information 2.

## Data Availability

All data analyzed in this study are included either in the published article or the Supplementary Information files.
