# Supplementary material for: Gait disorders induced by photothrombotic cerebellar stroke in mice
Source: Sci Rep. 2023 Sep 22;13:15805. doi: 10.1038/s41598-023-42817-4 (PMC10516889; doi:10.1038/s41598-023-42817-4)
Supplement: Supplementary file 1 — Supplementary Information 1. [file 41598_2023_42817_MOESM1_ESM.docx]

Gait disorders induced by photothrombotic cerebellar stroke in mice

Keisuke Inoue (inoue-keisuke248@g.ecc.u-tokyo.ac.jp)

affiliations

- Department of Life Sciences, Graduate School of Arts and Sciences, The University of Tokyo
- Department of Rehabilitation, JA Toride Medical Center

Meiko Asaka (meiko.asaka@riken.jp)

affiliation

- RIKEN center for Brain Science, Cognition and Behavior Joint Research Laboratory

Sachiko Lee（lee@met.nagoya-u.ac.jp）

affiliation

- Department of Rehabilitation Sciences, Graduate School of Medicine, Nagoya University

Kinya Ishikawa（pico.nuro@tmd.ac.jp）

affiliation

- The Center for Personalized Medicine for Healthy Aging, Tokyo Medical and Dental University.
- Department of Neurology and Neurological Science, Graduate School of Medical and Sciences, Tokyo Medical and Dental University.

*Dai Yanagihara (dai-y@idaten.c.u-tokyo.ac.jp)

affiliation

- Department of Life Sciences, Graduate School of Arts and Sciences, The University of Tokyo

RIKEN center for Brain Science, Cognition and Behavior Joint Research Laboratory

Supplementary Table S1. Numerical and statistical data on body weight, accelerating rotarod test, and ladder rung test.

|  |  |  | pre | post | two-way ANOVA | | |
| --- | --- | --- | --- | --- | --- | --- | --- |
|  |  |  |  |  | pre-post | group | interaction |
| Body weight (g) | | Stroke | 22.2±0.9 | 22.8±1.0 | F(1,10)=36.184 p<0.001 | F(1,10)=0.151 p=0.705 | F(1,10)=1.252 p=0.289 |
|  |  | Sham | 21.8±0.9 | 22.7±1.2 |  |  |  |
| Maximal latency of fall in accelerating rotarod test (s) | | Stroke | 137.2±42.2 | 178.5±35.5 | F(1,10)=9.879 p=0.010 | F(1,10)=0.738 p=0.410 | F(1,10)=0.274 p=0.612 |
|  |  | Sham | 144.2±33.9 | 202.0±50.7 |  |  |  |
| Number of steps  in ladder rug test (steps) | | Stroke | 135.2±14.6 | 146.5±19.3 | F(1,10)=1.868 p=0.202 | F(1,10)=0.007 p=0.933 | F(1,10)=1.097 p=0.320 |
|  |  | Sham | 139.5±5.5 | 141.0±14.0 |  |  |  |
| % missteps  in ladder rung test (%) | | Stroke | 11.0±2.1 | 13.5±1.3^＊‡^ | F(1,10)=0.010 p=0.923 | F(1,10)=16.205 p=0.022 | F(1,10)=10.495 p=0.009 |
|  |  | Sham | 10.4±2.0 | 8.1±1.7 |  |  |  |

Data were analyzed by repeated two-way ANOVA with a post-hoc Bonferroni test. Data are shown as means ± SD from six animals in each group. ＊ pre-test vs post-test, p < 0.05. ‡ sham vs stroke, p < 0.01.

Supplementary Figure S2. Horizontal ladder rung task apparatus.

Supplementary Table S3. Numerical and statistical temporal data for the right hindlimb in the treadmill walking test.

|  |  |  | pre | post | two-way ANOVA | | |
| --- | --- | --- | --- | --- | --- | --- | --- |
|  |  |  |  |  | pre-post | group | interaction |
| Right | |  |  |  |  |  |  |
| Stance duration (ms) | | Stroke | 134.1±2.0 | 130.6±5.4 | F(1,10)=2.618 | F(1,10)=0.307 | F(1,10)=0.020 |
|  |  | Sham | 131.6±4.0 | 127.4±3.7 | p=0.137 | p=0.592 | p=0.890 |
| Swing duration (ms) | | Stroke | 87.3±2.8 | 78.1±3.4 ＊† | F(1,10)=1.144 | F(1,10)=1.926 | F(1,10)=4.925 |
|  |  | Sham | 86.7±3.7 | 89.9±3.8 | p=0.310 | p=0.195 | p=0.051 |
| Cycle time (ms) | | Stroke | 221.3±2.6 | 208.7±8.3 | F(1,10)=2.685 | F(1,10)=0.123 | F(1,10)=1.959 |
|  |  | Sham | 218.3±7.0 | 217.3±6.0 | p=0.134 | p=0.734 | p=0.192 |
| Duty (%) | | Stroke | 60.5±1.0 | 62.4±0.7† | F(1,10)=0.034 | F(1,10)=3.517 | F(1,10)=6.030 |
|  |  | Sham | 60.3±0.8 | 58.7±1.1 | p=0.857 | p=0.090 | p=0.034 |
| Left | |  |  |  |  |  |  |
| Stance duration (ms) | | Stroke | 138.5±2.1 | 133.1±6.1 | F(1,10)=0.437 | F(1,10)=0.278 | F(1,10)=1.120 |
|  |  | Sham | 138.1±4.7 | 139.3±4.2 | p=0.523 | p=0.609 | p=0.315 |
| Swing duration (ms) | | Stroke | 83.8±3.3 | 76.1±3.1 | F(1,10)=3.292 | F(1,11)=0.012 | F(1,10)=0.771 |
|  |  | Sham | 80.8±4.2 | 78.2±2.6 | p=0.100 | p=0.915 | p=0.401 |
| Cycle time (ms) | | Stroke | 222.3±3.2 | 209.2±8.8 | F(1,10)=2.297 | F(1,10)=1.487 | F(1,10)=1.487 |
|  |  | Sham | 218.9±7.2 | 217.5±6.1 | p=0.161 | p=0.251 | p=0.251 |
| Duty (%) | | Stroke | 62.4±1.1 | 63.5±0.6 | F(1,10)=1.639 | F(1,10)=0.367 | F(1,10)=0.009 |
|  |  | Sham | 63.1±1.2 | 64.1±0.6 | p=0.229 | p=0.558 | p=0.926 |

Data were analyzed by repeated two-way ANOVA with a post-hoc Bonferroni test. The interaction in the right swing duration was close to significance (p = 0.051), suggesting cerebellar stroke shortened the swing duration. Data are shown as means ± SEM from six animals in each group. ＊ pre-test vs post-test, p < 0.05. † sham vs stroke, p < 0.05.
